# Ocular Albinism Type 1 Regulates Deltamethrin Tolerance in *Lymantria dispar* and *Drosophila melanogaster*

**DOI:** 10.3389/fphys.2019.00766

**Published:** 2019-06-19

**Authors:** Lili Sun, Peng Liu, Chenshu Zhang, Hui Du, Zhiying Wang, Timothy W. Moural, Fang Zhu, Chuanwang Cao

**Affiliations:** ^1^Key Laboratory of Sustainable Forest Ecosystem Management-Ministry of Education, School of Forestry, Northeast Forestry University, Harbin, China; ^2^Department of Entomology, Pennsylvania State University, University Park, PA, United States

**Keywords:** G-protein-coupled receptor, Asian gypsy moth, deltamethrin stress, cytochrome P450, physiological function

## Abstract

The ocular albinism type 1 (OA1), a pigment cell-specific integral membrane glycoprotein, is a member of the G-protein-coupled receptor (GPCR) superfamily that binds to heterotrimeric G proteins in mammalian cells. We aimed to characterize the physiological functions an insect OA1 from *Lymantria dispar* (*LdOA1*) employs in the regulation of insecticide tolerance. In the present study, we investigated the roles of *LdOA1* in response to deltamethrin exposure in both *L. dispar* and *Drosophila melanogaster*. *LdOA1* was expressed at the lowest level during the 4^th^ instar stage, while *LdOA1* was significantly upregulated in the 5^th^ instar and male stages. Knockdown of *LdOA1* by injecting dsRNA of *LdOA1* into gypsy moth larvae caused a 4.80-fold higher mortality than in control larvae microinjected with dsRNA of *GFP* under deltamethrin stress. Nine out of 11 *L. dispar CYP* genes were significantly downregulated under deltamethrin stress in *LdOA1* silenced larvae as compared to control larvae. Moreover, the *LdOA1* gene was successfully overexpressed in *D. melanogaster* using transgenic technique. The deltamethrin contact assay showed that the *LdOA1* overexpression in flies significantly enhanced the tolerance to deltamethrin compared to the control flies. Furthermore, the downstream *Drosophila* CYP genes were upregulated in the *LdOA1* overexpression flies, suggesting *LdOA1* may play a master switch role in P450-mediated metabolic detoxification. This study is the first report of an insect OA1 gene regulating insecticide tolerance and potentially playing a role in the regulation of downstream cytochrome P450 expression. These results contribute to the future development of novel insecticides targeting insect GPCRs.

## Introduction

The Asian gypsy moth, *Lymantria dispar* (Lepidotera: Erebidae), is a key forest pest mainly present in Asia and Europe. As a polyphagous herbivore, *L. dispar* can feed on at least 500 species of plants in over 100 botanical families, particularly on oak, poplar, and birch (Roy et al., 1995; Lazarevic et al., 1998). Outbreaks of *L. dispar* lead to serious damage of forests, orchards, and landscaping. In Northern and Eastern China, the Asian gypsy moth has defoliated about two million acres of forest each year over the past 20 years (Sun et al., 2014). Since the last century, the Asian gypsy moth has also been observed in North America and has presented a huge threat to North American forests once the population became established (Roy et al., 1995; United States Department of Agriculture [USDA], 2016). Compared to the subspecies of gypsy moth known in Europe and North America, the “Asian form” gypsy moth has active flying females and a much broader host range (Roy et al., 1995). To manage gypsy moths, a variety of strategies have been used, including chemical control, release of natural enemies (*Coccygomimus disparis*), biological control (*Bacillus thuringiensis*), and host plant transgenic engineering (transgenic poplar expressing fusion protein genes of the spider insecticidal peptide and *Bt*-toxin C-peptide) (Hou et al., 2009; Cao et al., 2010; Li, 2012). Among these strategies, spraying insecticides (e.g., organophosphates, pyrethroids, and carbamates) has remained the most rapid and effective control method in China so far (Ni et al., 2009; Li, 2012). However, it has become increasingly important to develop new insecticides with safe and novel modes of action due to the growing challenges associated with the development of insecticide resistance, off-target effects, and environmental contamination (Audsley and Down, 2015).

G-protein-coupled receptors (GPCRs) comprise one of the largest and most diverse family of membrane proteins, which transduce extracellular signals into cellular responses to hormones, neurotransmitters, and environmental stimulants (Hill et al., 2002; Rosenbaum et al., 2009). The ligands of GPCRs are extremely diverse, including chromophores, neuropeptides and hormones, acetylcholine, biogenic amines, nucleotides and nucleosides, lipids and eicosanoids, olfactory and taste substances, and so on (Broeck, 2001; Klabunde and Hessler, 2002). Ligand binding triggers GPCRs conformational changes, activating complex cytosolic signaling networks and causing intracellular responses. Therefore, GPCRs play various important roles in modulating sense of vision, smell and taste, immune system and autonomic nervous system, regulating reproduction, behavior, osmoregulation, growth, and development, and they draw much attention for drug discovery (Klabunde and Hessler, 2002; Garland, 2013). As a result, GPCRs are targets for many of the best-selling drugs and roughly 30–50% of all medicines in the pharmaceutical market (Klabunde and Hessler, 2002; Filmore, 2004; Garland, 2013).

Insect GPCRs have been recognized as highly attractive targets for new generation insecticide discovery due to their critical functions in insect reproduction, development, behavior, and metabolism (Broeck, 2001; Bai et al., 2011; Caers et al., 2012; Meyer et al., 2012; Audsley and Down, 2015; Bai and Palli, 2016; Hill et al., 2018). Inhibition or overstimulation of insect GPCRs may cause the death of a pest or disrupt its normal physiological processes and reduce pest populations (Mitri et al., 2009; Bai et al., 2011; Caers et al., 2012). Ocular albinism type 1 protein (OA1; *GPR143*) is a pigment cell-specific glycoprotein with the characteristic seven transmembrane structural features, and amino acid sequence conservation consistent with the GPCR family of membrane proteins. In humans and mice, OA1 mutations have been documented to generate a decrease or deficiency in melanin biogenesis, which often results in ocular albinism (d′Addio et al., 2000). The ocular albinism disease causes severe reductions in visual acuity via an increase of retinal pigment loss by the OA1 mutation (Schiaffino, 2010). Insect melanin plays a key role in pigment synthesis. For example, pigmentation is one of the most variable traits in the genus *Drosophila* (Wittkopp et al., 2003). However, the functions and mechanisms of insect OA1 in response to insecticide stresses remain unclear. Our previous study reported the transcriptional expression patterns of *LdOA1* under deltamethrin, carbaryl, and omethoate stresses (Cao et al., 2014). Here, we further investigated whether the *LdOA1* is associated with tolerance to insecticide stresses and involved in the regulation of downstream detoxification genes (e.g., cytochrome P450s) in response to insecticide stresses.

## Materials and Methods

### Insects

*Lymantria dispar* eggs and the artificial diet for larvae were purchased from the Research Institute of Forest Ecology, Environment and Protection, Chinese Academy of Forestry (Beijing, China). The *L. dispar* line purchased had been maintained in the lab for >5 years without exposure to pesticides. Rearing of *L. dispar* was modified from the protocol of Cao et al. (2015). In brief, gypsy moth larvae were kept in transparent plastic bottles (250 mL) with artificial diets at 25 ± 1°C and 16:8 h light:dark photoperiod. The humidity of the rearing bottles and artificial diets was maintained with botanical sponges soaked with water. Healthy gypsy moth larvae of 3^rd^ instar larvae were used for bioassays, dsRNA injection, and RNA extractions.

### RNA Extraction, cDNA Synthesis, and qRT-PCR Analysis

Total RNA was extracted from insect samples with Trizol reagent (Invitrogen®) following the manufacturer’s protocol. The total RNA was treated with DNase I (Ambion Inc., Austin, TX, United States) for contaminating genomic DNA (gDNA) removal. Approximately 0.5 μg of DNase I-treated RNA was reverse transcribed to cDNA using 1 μM of oligo (dT) primer in a 10 μL reaction. Synthesized cDNA was diluted with sterile water to 100 μL, and the solution was used as templates for qRT-PCR in an MX3000P machine (StrataGene, Agilent, CA, United States). *Actin* (MK926773), *EF1α* (MK926771), and *TUB* (MK926772) genes were used as reference genes for gypsy moths and *RpL32* and *ATP Binding Protein* (*ABP*) genes were chosen as internal controls for *Drosophila* (Willis et al., 2010). The reaction mixture (20 μL) contained 10 μL of SYBR Green real-time PCR master mix (Toyobo, Osaka, Japan), 0.5 μM each of forward and reverse primers, and 2 μL of cDNA template (equivalent to 100 ng of total RNA). DNA amplification was conducted using the following cycling parameters: 94°C for 30 s followed by 45 cycles of 94°C for 12 s, 60°C for 30 s, 72°C for 40 s, and 1 s at 82°C for plate reading. A melting curve generated for each sample at the end of each run was used to assess the purity of the amplified products. Expression levels were calculated using the 2^–ΔΔCT^ method (Pfaffl et al., 2002). qRT-PCR was performed with three independent biological repeats using 20 *Drosophila* or gypsy moth larvae for each replicate to ensure reproducibility.

### RNA Interference (RNAi) Bioassay in *L. dispar* Larvae

The function of the *LdOA1* gene was investigated using the RNA interference (RNAi) technique. Approximately 200–650 bp fragments based on the full length of the *LdOA1* gene and a green fluorescent protein gene (GFP-pMW1650, a generous gift from Professor Nannan Liu, Auburn University, Alabama, United States) were used to generate cDNA of the *L. dispar* larvae and the pMW1650 plasmid as templates, respectively. The specific primers were designed with the T7 promoter (Supplementary Table S1). dsRNA was synthesized *in vitro* using the MEGAscript T7 High Yield Transcription kit (Ambion) following the manufacturers’ protocol. dsRNA was purified with phenol/chloroform followed by ethanol precipitation. dsRNA was diluted with nuclease-free water, and a 1∼2 μg/μL dsRNA solution (0.5∼1 μL) was microinjected into the penultimate posterior abdominal section of each 3^rd^ instar gypsy moth larvae using an injection needle (MICROLITER^TM^ #65 with 33-gauge needle, Hamilton Co., Reno, NV, United States) under CO_2_ gas exposure. Control gypsy moth larvae were microinjected with the GFP dsRNA. Injected gypsy moth larvae were removed to recover for 2 h at room temperature and then were reared on an artificial diet under a 16:8 light:dark photoperiod at 25 ± 1°C. After 48 h, 72 h, 96 h, and 120 h, the *L. dispar* larvae were selected to measure effects of dsRNA microinjection on *LdOA1* transcript levels by qRT-PCR.

### Deltamethrin Challenge in *L. dispar* Larvae

To study the effects of deltamethrin on cytochrome P450 genes in *L. dispar* larvae microinjected with dsRNAs of *LdOA1* or *GFP* (ds*LdOA1* or ds*GFP*), LC_20_ value after 24 h of deltamethrin (15 mg/L) exposure was used as the treatment concentration (Sun et al., 2016). 15 mg/L of deltamethrin was added to the artificial diet and fed to the 3^rd^ instar larvae at 120 h after microinjection with ds*LdOA1* and ds*GFP*. 20 healthy larvae were collected for each replicate at 24 h and stored at -80°C for RNA extraction. Each treatment was repeated three times. The cumulative mortality of larvae in both control (ds*GFP*) and treatment (ds*LdOA1*) was recorded 5 days after the deltamethrin challenge. The transcript levels of *L. dispar CYP* genes were measured using qRT-PCR. The primers are listed in Supplementary Table S1.

### Construction of *UAS-LdOA1* Transgenic *Drosophila*

The *Drosophila* expressing *LdOA1* gene was generated with the GAL4/UAS system following the protocol of Riveron et al. (2014). Briefly, the full-length *LdOA1* was amplified from cDNA using RT-PCR with the primers containing restriction sites for *EcoR*I and *Xho*I listed in Supplementary Table S1 and ligated into pMD18-T vector. After digesting plasmids LdOA1/pMD18-T and pUAST-attB with *EcoR*I and *Xho*I, the *LdOA1* gene was subsequently cloned into the *pUAST-attB* vector to obtain a recombinant plasmid, *pUAST-attB-LdOA1*. Using PhiC31 system (Markstein et al., 2008), the recombinant plasmid *pUAST-attB-LdOA1* was microinjected into the embryos of *D. melanogaster* strain carrying docking site on chromosome 3 [UAS-phi2b2a; VK5] to generate the transformant line UAS-LdOA1. To ubiquitously express *LdOA1* in *Drosophila*, UAS-LdOA1 flies were crossed with a ubiquitous tissue driver Act5C-Gal4. UAS-LdOA1 crossing with w1118 was used as the control. To confirm the expression of *LdOA1* in the transformant *Drosophila*, the total RNA and DNA were extracted from three pools of ten flies of each strain. The *LdOA1* gene was amplified by RT-PCR or PCR.

### *Drosophila* Contact Bioassay and Deltamethrin Challenge

The contact bioassay was used to measure the susceptibility of *Drosophila* to deltamethrin. 200 μL of acetone was used as a control, and different concentrations of deltamethrin diluted in acetone were coated on the inside of glass scintillation vials (40 mL) by rolling the vial until the acetone evaporated following the protocol described in our previous study (Zhu et al., 2010). The small diet (approximately 1 cm × 1 cm) was placed into vials plugged with cotton balls. Ten 1–3 day(s) old posteclosion *Drosophila* adults (sex ratio = 1:1) were placed into each vial, and the mortality was scored after 24 h of treatment. Each concentration was repeated three times independently. *Drosophila* were reared under a 16:8 light:dark photoperiod at 25 ± 1°C. The medium lethal concentration (LC_50_) and 20% lethal concentration (LC_20_) values at 24 h obtained from strains (Act5 > UAS-LdOA1 and UAS-LdOA1 > w^1118^) were calculated by POLO software (LeOra Software Inc., Petaluma, CA, United States). Each treatment was repeated three times independently. The transcriptional expression levels of *Drosophila* cytochrome P450 genes in UAS-LdOA1 > w^1118^ and Act5 > UAS-LdOA1 strains were measured by qRT-PCR. Before the bioassay, 20 control (UAS-LdOA1 > w^1118^) and 20 *LdOA1* overexpression (Act5 > UAS-LdOA1) flies (sex ratio = 1:1) were selected to measure the weight. Three replicates were performed. The average weight of each fly in both groups was calculated and compared.

### Statistical Analysis

The data are represented in figures as means ± SD. The difference between two treatments was compared by Student’s *t-*test (two-tailed paired *t*-test). The difference among multiple samples was calculated by one-way analysis of variance (ANOVA) followed by Student-Newman-Keuls multiple comparisons test using a statistical software package (GraphPad InStat version 3.05). The level of significance was set at *P* < 0.05.

## Results

### Developmental Expression Profile of the *LdOA1* Gene

**FIGURE 1:**
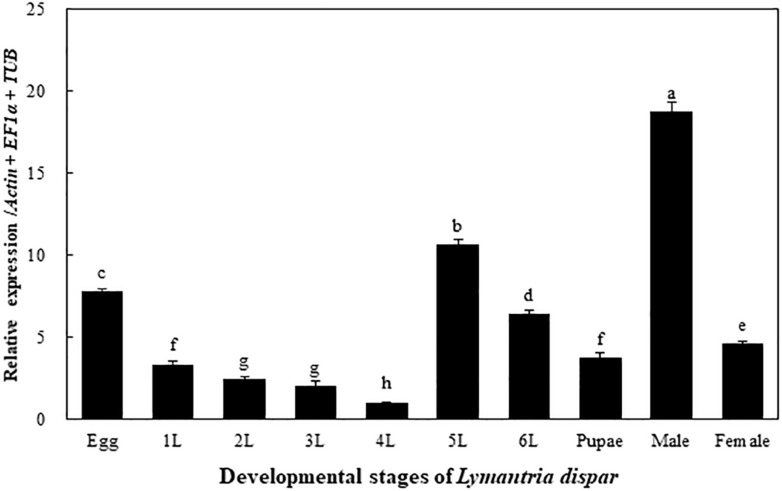
Transcriptional expression of the *LdOA1* gene during different developmental stages of *L. dispar*. 1 to 6 L represent 1st to 6^th^ instar *L. dispar* larvae. Data were normalized to three most stable reference genes *Actin*, *EF1α*, and *TUB*. The 2^–ΔΔCT^ method was used to calculate relative expression levels. The expression was shown as mean ± SD from three independent assays. Different lowercase letters above bars indicate significant difference among developmental stages according to ANOVA followed by Student-Newman-Keuls multiple comparisons test (*P* < 0.05).

The transcriptional expression levels of the *LdOA1* gene were examined in different developmental stages of the Asian gypsy moth, including eggs, larvae (1^st^ to 6^th^ instars), pupae, male and female adults. The expression of the *LdOA1* gene varies among different developmental stages. Compared with other stages, the expression of *LdOA1* in the 4^th^ instar larva exhibited the lowest level. Whereas, the expression level of *LdOA1* in the male was the maximum (Figure 1).

### *LdOA1* Silencing and Its Response to Deltamethrin Challenge

**FIGURE 2:**
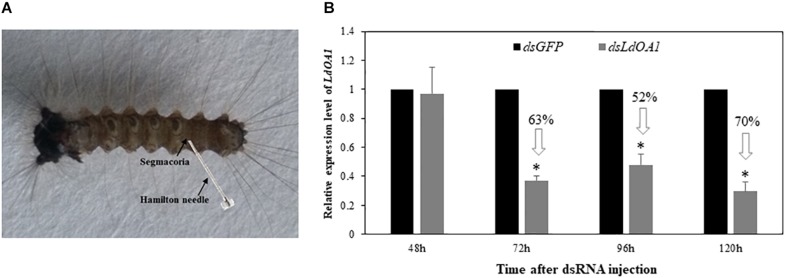
Effect of RNAi on *LdOA1* gene expression in 3^rd^ instar *L. dispar* larvae after dsRNA injection. **(A)** 3^rd^ instar *L. dispar* larva receiving dsRNA injection via the segmacorla. **(B)** *LdOA1* transcript levels were determined by qRT-PCR for green fluorescent protein dsRNA-injected (ds*GFP*) and *LdOA1* dsRNA-injected (ds*LdOA1*) 3^rd^ instar *L. dispar* larvae. Data were normalized to three most stable reference genes *Actin*, *EF1α*, and *TUB*. The 2^–ΔΔCT^ method was used to calculate relative expression levels. The gene expression was shown as mean ± SD from three independent assays. Asterisk (^*^) indicates significant difference according to Student’s *t*-test (*P* < 0.05).

To investigate the function of *LdOA1* in response to insecticide challenging, we first employed RNA interference (RNAi) technology to knock down the expression of *LdOA1* in gypsy moth by injecting dsRNA into the penultimate posterior abdominal section of the 3^rd^ instar larva (Figure 2A). RNAi results showed that the mRNA level of *LdOA1* in the 3^rd^ instar larvae microinjected with ds*LdOA1* was significantly lower than that in the larvae microinjected with ds*GFP* (Figure 2B). At 120 h after injection of ds*LdOA1*, the *LdOA1* mRNA level decreased by 70% (Figure 2B).

**FIGURE 3:**
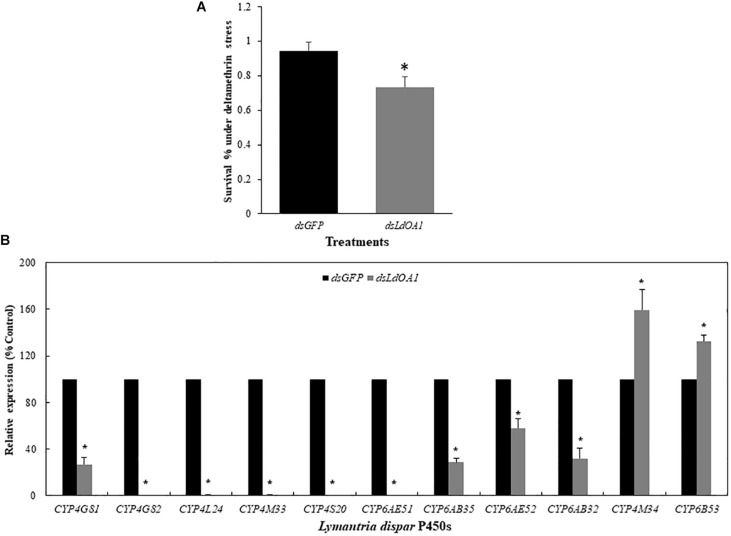
Effects of sublethal deltamethrin (24 h LC_20_ = 15 mg/L) on 3^rd^ instar *L. dispar* larvae survival **(A)** and *CYP* gene expressions **(B)** 120 h after injecting dsRNA of green fluorescent protein (*GFP*, control) or dsRNA of *LdOA1*. The cumulative mortality of larvae in both control and treatment was recorded 5 days after the deltamethrin challenge. Steady-state *CYP* transcript levels were determined by qRT-PCR for *GFP* dsRNA-injected (*dsGFP*) and *LdOA1* dsRNA-injected (ds*LdOA1*) 3^rd^ instar *L. dispar* larvae. Data were normalized to *Actin*, *EF1α*, and *TUB* internal controls, and 2^–ΔΔCT^ method was used to obtain relative expression levels, expressed as mean ± SD from assays performed in triplicate. Asterisk (^*^) indicates significant differences among different treatments at the same gene by Student’s *t*-test (*P* < 0.05).

Gypsy moth larvae with silenced *LdOA1* or *GFP* were then treated with the sublethal dose of deltamethrin (LC_20_ = 15 mg/L). Under the stress of deltamethrin, larvae treated with ds*LdOA1* showed a 4.80-fold greater mortality than the larvae treated with ds*GFP* (Figure 3A).

Eleven cytochrome P450 genes were selected from the transcriptome of gypsy moth and our previous studies (Sun et al., 2014; Cao et al., 2015) to detect the effects of *LdOA1* RNAi and deltamethrin challenge on the expression of down-stream cytochrome P450 genes (Figure 3B). Except for *CYP4M34* and *CYP6B53*, the expression of nine cytochrome P450 genes was significantly decreased under deltamethrin stress in *LdOA1* RNAi larva compared to the control larva (Figure 3B). These results showed that the *LdOA1* silencing decreased the tolerance to deltamethrin in *L. dispar* larva and suppressed cytochrome P450 gene expression under the stress of deltamethrin.

### *Drosophila* With Ectopically Expressed *LdOA1* Shows Enhanced Tolerance to Deltamethrin

To further investigate the function of *LdOA1* in the regulation of insecticide stress tolerance, the *LdOA1* was ectopically expressed in *Drosophila* using GAL4/UAS system. After confirming the expression of LdOA1 in F1 progeny by RT-PCR and PCR (Supplementary Figure S1), the results of deltamethrin toxicity assay were shown in Table 1. The LC_50_ values of deltamethrin for the control flies (UAS-LdOA1 > w^1118^) and *LdOA1* overexpressed flies (Act5 > UAS-LdOA1) were 3.97 and 8.03 mg/L, respectively (Table 1), revealing that there was a 2.02-fold higher tolerance to deltamethrin in the flies with ectopically overexpressed *LdOA1* gene. The 95% confidence intervals (95% CI) were not overlapped between the *LdOA1* overexpression flies and control flies, suggesting the enhancement of deltamethrin tolerance was significant (Table 1).

**TABLE 1:** Deltamethrin toxicity on *LdOA1* overexpression and control flies.

**Strain**		**Number**	**LC_50_ (95% CI) mg/L**	**LC_20_ (95% CI) mg/L**	**Slope ± SEM**	**χ^2^**	***df***
Control	UAS-LdOA1 > w^1118^	240	3.97 (3.19–4.79)	1.94 (1.36–2.49)	2.70 ± 0.31	5.93	19
*LdOA1* overexpression	Act5 > UAS-LdOA1	240	8.03 (5.11–12.40)	4.08 (3.01–5.02)	2.86 ± 0.37	16.56	19

### Expression of Cytochrome P450 Genes in *LdOA1*-Expressing *Drosophila*

**FIGURE 4:**
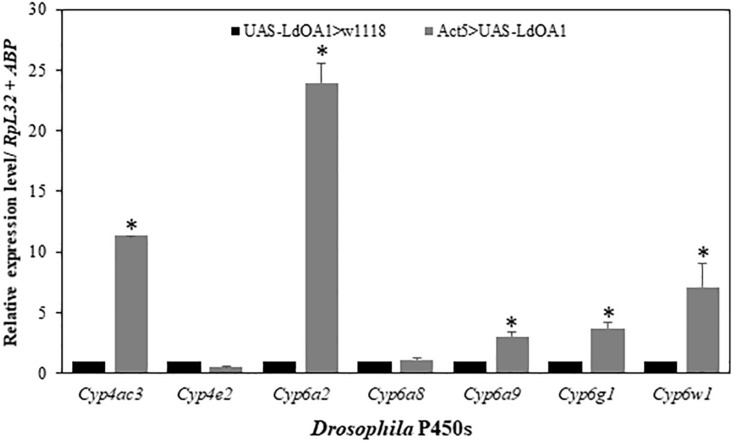
The relative expression levels of *Drosophila CYP* genes in the *LdOA1* overexpression flies (Act5 > UAS-LdOA1) comparing to the control flies (UAS-LdOA1 > w1118). Data were normalized to two most stable reference genes *RpL32* and *ABP*. The 2^–ΔΔCT^ method was used to calculate relative expression levels. The gene expression was shown as mean ± SD from three independent assays. ^*^ above bars indicate significant difference according to Student’s *t*-test (*P* < 0.05).

To investigate the regulation of *LdOA1* on *Drosophila* cytochrome P450 gene expression in *LdOA1*-expressing *Drosophila*, the expressions of seven *Drosophila* cytochrome P450 genes were examined and compared in *LdOA1*-expressing *Drosophila* and control flies (Figure 4). Among the seven P450 genes, five of them, including *Cyp4ac3*, *Cyp6a2*, *Cyp6a9, Cyp6g1*, and *Cyp6w1*, were upregulated in the *LdOA1*-expressing *Drosophila* compared to the control flies (Figure 4). The expression levels of the remaining two *Drosophila CYP* genes (*Cyp4e2* and *Cyp6a8*) exhibited no difference between *LdOA1* overexpression files and control files (Figure 4).

## Discussion

LdOA1 GPCR is primarily associated with intracellular late endosome/lysosome and melanosomes, with its N-terminus located toward the lumen of the organelle and its C-terminus toward the cytoplasm (d′Addio et al., 2000). The OA1 has been observed to provide the cell with a mechanism to sense melanosome maturation and regulate organelle biogenesis and homeostasis (Raposo and Marks, 2007; Bai et al., 2014). In humans and mice, the *OA1* genes encode for pigment cell-specific integral membrane glycoproteins that contain several putative transmembrane domains, and consist of 404 and 405 amino acids, respectively. The *OA1* gene is exclusively expressed in the pigment cells of the skin and eye (Bassi et al., 1996; Schiaffino et al., 1996, 1999). To date, the first component of the signaling cascade triggered by OA1 has been identified as Gαi3 (Young et al., 2008, 2011, 2013). However, identity of its downstream targets and the cause of macromelanosome production remain unknown. Recently, Cao et al. (2014) reported that the expression of *LdOA1* in 3^rd^ instar *L. dispar* larvae was significantly inhibited by deltamethrin, carbaryl and omethoate stresses during the 72 h period of exposure. Sun et al. (2016) had provided additional confirmation that knockdown of the *LdOA1* gene not only increased the mortality of 3^rd^ instar *L. dispar* larvae but also decreased their body weight, relative consumption rate (RCR), and approximate digestibility (AD) based on the nutrition utilization index. In the present study, we investigated the roles of *LdOA1* in the regulation of deltamethrin tolerance and cytochrome P450 expression in both *L. dispar* and *D. melanogaster*. Our study showed that RNAi of *LdOA1* in *L. dispar* larvae led to significantly decreased deltamethrin tolerance (Figure 3A). In contrast, the overexpression of *LdOA1* in *Drosophila* resulted in enhanced tolerance to deltamethrin stress over control flies (Table 1). These results suggest that *LdOA1* plays an important role in the regulation of deltamethrin tolerance. Similarly, in *Culex pipiens pallens*, several GPCRs have shown the functions associated with deltamethrin resistance (Hu et al., 2007; Sun et al., 2012; Zhou et al., 2018). In *C. quinquefasciatus*, four GPCR-related genes are involved in the regulation of permethrin resistance (Liu et al., 2007; Li et al., 2015). In *Tribolium castaneum*, RNAi of a GPCR *Latrophilin* decreases larval tolerance to carbamates and organophosphates, suggesting the role of *Latrophilin* in insecticide tolerance (Gao et al., 2018). These evidence indicate that GPCR regulatory pathways are a common mechanism in the regulation of insecticide tolerance or resistance (Liu, 2015).

Our study also reveals that *LdOA1* may play a master switch role for P450-mediated metabolic detoxification. Under deltamethrin stress, the expressions of nine *L. dispar* P450 genes (*CYP4G81*, *CYP4G82*, *CYP4L24*, *CYP4M33*, *CYP4S20*, *CYP6AE51*, *CYP6AB35*, *CYP6AE52*, and *CYP6AB32*) in gypsy moth larva with *LdOA1* silencing was significantly decreased compared to the control larva (Figure 3B). In *LdOA1* overexpression *Drosophila*, five *Drosophila* P450 genes (*Cyp4ac3*, *Cyp6a2*, *Cyp6a9, Cyp6g1*, and *Cyp6w1*) were significantly upregulated compared to control flies (Figure 4). Some of these genes (e.g., *Cyp6a2*, *Cyp6g1*, and *Cyp6w1*) have been shown to confer insecticide resistance or link with xenobiotic metabolism in *Drosophila* populations (Daborn et al., 2002; Battlay et al., 2016; Denecke et al., 2017; Seong et al., 2018; Huang et al., 2019). Our study is consistent with other insect GPCR studies. For example, in *C. pipiens pallens*, the expressions of *CYP6A1* and opsin were upregulated in mosquito cells transfected with arrestin GPCR and suppressed after arrestin silencing (Sun et al., 2012). Knockdown of a rhodopsin-like GPCR gene or its downstream effectors from permethrin resistant *C. quinquefasciatus* reduced the expression of four permethrin-resistance related P450 genes (Li et al., 2014, 2015; Li and Liu, 2017, 2018). A recent study showed that knockdown of an opsin GPCR, NYD-OP7, from permethrin resistant *C. pipiens pallens* strain led to reduced expression of five P450 genes from seven genes tested (Zhou et al., 2018).

Cytochrome P450s are extremely important detoxification enzymes and play vital roles in xenobiotic adaptation (Li et al., 2007; Liu and Zhu, 2011; Feyereisen, 2012; Zhu et al., 2016; Zhang et al., 2018). Cytochrome P450-mediated detoxification is a universal mechanism contributing resistance to various pesticides and nature-derived toxins, regardless of the mode of action (Scott et al., 1998; Feyereisen, 2012; Zhu et al., 2013). Therefore, understanding the genetic machinery underpinning regulation of P450-mediated xenobiotic detoxification will facilitate the development of new pest control strategies (Misra et al., 2011; Zhu et al., 2014). Insect nuclear receptors play important roles in the regulation of xenobiotic genes induction (Fahrbach et al., 2012). Recent studies illustrated that an evolutionary conserved Cap’n’collar isoform C (CncC)/Keap1 pathway plays a role in the xenobiotic responses in *Drosophila* as well as agricultural and urban pests (Misra et al., 2011, 2013; Kalsi and Palli, 2017a, b). Here, we identified a GPCR gene from the Asian gypsy moth, *LdOA1*, that was involved in the regulation of deltamethrin tolerance potentially through regulating downstream cytochrome P450 genes. However, further experiments are required to investigate whether *LdOA1*-mediated regulatory pathway directly regulates the expression of cytochrome P450 genes or not. Insect *OA1* can serve as a very promising target for more effective pest control. In general, insect GPCRs may therefore be a valuable target for the development of new insecticides.

## Data Availability

The datasets generated for this study can be found in the NCBI, Actin (MK926773), EF1α (MK926771), and TUB (MK926772).

## Author Contributions

CC, FZ, and ZW conceived and designed the experimental study. LS, PL, CZ, and HD conducted the experiments. CC, FZ, and TM analyzed the data and drafted the manuscript.

## Conflict of Interest Statement

The authors declare that the research was conducted in the absence of any commercial or financial relationships that could be construed as a potential conflict of interest.

## Supplementary Material

FIGURE S1 The expression of *LdOA1* gene was shown in lanes 1 and 2 (Act5 > UAS-LdOA1) but not in lanes 3 and 4 (UAS-LdOA1 > w^1118^) on 1% agarose gel.

TABLE S1 Primers used for dsRNA synthesis, transformant *Drosophila* and qRT-PCR.
