# Supplementary figures and images for: Ocular Albinism Type 1 Regulates Deltamethrin Tolerance in Lymantria dispar and Drosophila melanogaster
Source: Front Physiol. 2019 Jun 19;10:766. doi: 10.3389/fphys.2019.00766 (PMC6594220; doi:10.3389/fphys.2019.00766)

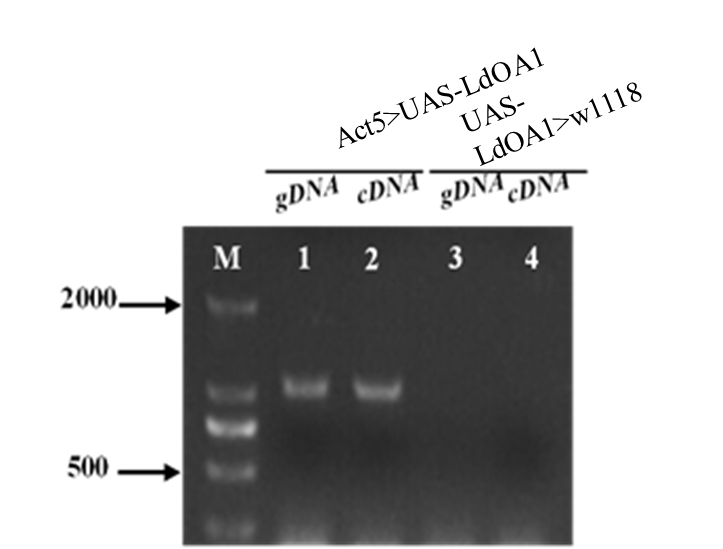

Supplement: FIGURE S1 — The expression of LdOA1 gene was shown in lanes 1 and 2 (Act5 > UAS-LdOA1) but not in lanes 3 and 4 (UAS-LdOA1 > w1118) on 1% agarose gel. [file Image_1.TIF]
