# Supplementary material for: Ocular Albinism Type 1 Regulates Deltamethrin Tolerance in Lymantria dispar and Drosophila melanogaster
Source: Front Physiol. 2019 Jun 19;10:766. doi: 10.3389/fphys.2019.00766 (PMC6594220; doi:10.3389/fphys.2019.00766)
Supplement: TABLE S1 — Primers used for dsRNA synthesis, transformant Drosophila and qRT-PCR. [file Table_1.DOCX]

**Table S1. Primers used for dsRNA synthesis, transformant *Drosophila* and qRT-PCR.**

| **Gene** | **Purpose Sequence（5’ to 3’）** | |
| --- | --- | --- |
| *LdOA1* | qRT-PCR | F: GTTCTATCTTTGCTGGTTGCCG |
|  |  | R: ATTGTAGCCTCGTGGGTTCTGA |
| *Actin* | qRT-PCR | F: AGAAGCACTTGCGGTGGACAAT |
|  |  | R: ACCTGTACGCCAACACTGTCAT |
| *EF1α* | qRT-PCR | F: TTTGCCTTCCTTGCGCTCAACA |
|  |  | R: TGTAAAGCAGCTGATCGTGGGT |
| *TUB* | qRT-PCR | F: AATGCAAGAAAGCCTTGCGCCT |
|  |  | R: ATGAAGGAGGTCGACGAGCAAA |
| *CYP4G81* | qRT-PCR | F: CCTACCAGAAAAGTCTGCCAATCG |
|  |  | R: CACTTTGGCTATTTTACGGGACTG |
| *CYP4G82* | qRT-PCR | F: GACCGACCGGCCACATTTTATG |
|  |  | R: GTTCCGGATGTCTATGGATGCC |
| *CYP4L24* | qRT-PCR | F: ACCCAGACTTGTATGAAGATCCTC |
|  |  | R: GTGTTAGTTCCGCAGACATCTC |
| *CYP4M33* | qRT-PCR | F: GCCGCAATTTGAAAGAAACCACAG |
|  |  | R: CCTATGCAATTTCTGGGTCCAG |
| *CYP4S20* | qRT-PCR | F: CGTGTTAGTGAAGAAAGGCACC |
|  |  | R: CGGAACGAGCTTGAAGTTACGA |
| *CYP6AE51* | qRT-PCR | F: GAAGGACCCAGAATGTGTATAGG |
|  |  | R: GGGCATCTCATGTGCTAACT |
| *CYP6AB35* | qRT-PCR | F: GGCTGAGAAGACCGATAGAAAC |
|  |  | R: CGTAGCGATCATAGCGATTGT |
| *CYP6AE52* | qRT-PCR | F: ACATGTGAGAGTCCACAAGAAA |
|  |  | R: TCTGGTGATTGCACGATAAGAA |
| *CYP6AB32* | qRT-PCR | F: CGAGCTGGTGAAGCGTAAA |
|  |  | R: ACAAGCCCTCTGGTAGAAATG |
| *CYP4M34* | qRT-PCR | F: GGAAAGCTGGTGTTAGAATTTTCC |
|  |  | R: CTGCAACATTCGCTAAGACGTATG |
| *CYP6B53* | qRT-PCR | F: GATCTGTGCCGGGAACTTTAT |
|  |  | R: TTTCTACGTGGGCGGTTATG |
| *LdOA1* | dsRNA synthesis | F: TAATACGACTCACTATAGGGATGATTGTGAACCCAATTATGT  R: TAATACGACTCACTATAGGGCAAAGTTGAATAATTATTGATT |
| *GFP* | dsRNA synthesis | F: TAATACGACTCACTATAGGGGGAGAAGAACTTTTCACTGG |
|  |  | R: TAATACGACTCACTATAGGGAGTTGAACGGATCCATCTTC |
| *LdOA1* | Transformed *Drosophila* | F: CCGGAATTCATGATTGTGAACCCAATTAT  R: CTAGCTCGAGCAAAGTTGAATAATTATTGA |
| *Cyp4ac3* | qRT-PCR | F: CCCGAAACCAAATCAATTCCTGCC  R: GCTGAGTGCGCAGTACGATTCCG |
| *Cyp4e2* | qRT-PCR | F: GAGCTCGTCTCTAAGGATGGCTA |
|  |  | R: GTATCTGTGGGGACGGCATACC |
| *Cyp6a2* | qRT-PCR | F: AGCGCTTCTCGCCGGAGAAAGT |
|  |  | R: CATGGGAGCAACGAATATGGAGG |
| *Cyp6a8* | qRT-PCR | F: GTTCTCCACGTGCTCGAAGACG  R: GATAGATCAATGGCCATGAGGACC |
| *Cyp6a9* | qRT-PCR | F: GCAGGCCAGAAGTGGATTGGC  R: CCACAAGCATGCTACTGCGATTAG |
| *Cyp6g1* | qRT-PCR | F: GAGCGCTCCATCGCTCCAATGA  R: AGCTGCTAGCTGCTCAGCACGC |
| *Cyp6w1* | qRT-PCR | F: GGACACTGTCCAGCACAACGGG  R: CAGATGCAGTTGCAGTTGCACCC |
| *RpL32* | qRT-PCR | F: GGCCCAAGATCGTGAAGAAGCG  R: CAGCTCGCGCACGTTGTGC |
| *ABP* | qRT-PCR | F: GTCCGCCAATACGGAGTTCTTC  R: CCCAGCTCCTGTTTAATGACAGC |
